# Do ethnobotanical and laboratory data predict clinical safety and efficacy of anti-malarial plants?

**DOI:** 10.1186/1475-2875-10-S1-S7

**Published:** 2011-03-15

**Authors:** Merlin Willcox, Françoise Benoit-Vical, Dennis Fowler, Geneviève Bourdy, Gemma Burford, Sergio Giani, Rocky Graziose, Peter Houghton, Milijaona Randrianarivelojosia, Philippe Rasoanaivo

**Affiliations:** 1 Research Initiative on Traditional Antimalarial Methods (RITAM), 66 Lye Valley, Oxford OX3 7ER, UK; 2 Department of Primary Health Care, University of Oxford, UK; 3 CNRS; LCC (Laboratoire de Chimie de Coordination) UPR8241; 205, route de Narbonne, F-31077 Toulouse, France and Université de Toulouse III ; UPS, LCC; 118, route de Narbonne, F-31077 Toulouse, France; 4 Service de Parasitologie-Mycologie, Centre Hospitalier Universitaire de Toulouse, TSA 50032, 31059 Toulouse cedex 9, France et Faculté de Médecine de Rangueil, Université de Toulouse III, UPS, 31059 Toulouse, France; 5 Université de Toulouse, UPS; UMR152 (Laboratoire de Pharmacochimie des Substances Naturelles et Pharmacophores Redox), F-31062 Toulouse, Cedex 9, France; 6 Institut de Recherche pour le Développement, UMR152, F-31062 Toulouse cedex 9, France; 7 Aang Serian Community College, PO Box 19, Monduli, Arusha, Tanzania; 8 Aidemet ONG, Bamako, Mali; 9 Rutgers University, NJ, USA; 10 Dept of Pharmacy, King’s College London, Franklin-Wilkins Building, 150 Stamford Street, LONDON SE1 8WA, UK; 11 Unité de Recherche sur le Paludisme, Institut Pasteur de Madagascar, Antananarivo, Madagascar; 12 Laboratoire de Biothérapeutique, Institut Malgache de Recherches Appliquées, BP 3833, 101-Antananarivo, Madagascar

## Abstract

**Background:**

Over 1200 plant species are reported in ethnobotanical studies for the treatment of malaria and fevers, so it is important to prioritize plants for further development of anti-malarials.

**Methods:**

The “RITAM score” was designed to combine information from systematic literature searches of published ethnobotanical studies and laboratory pharmacological studies of efficacy and safety, in order to prioritize plants for further research. It was evaluated by correlating it with the results of clinical trials.

**Results and discussion:**

The laboratory efficacy score correlated with clinical parasite clearance (r_s_=0.7). The ethnobotanical component correlated weakly with clinical symptom clearance but not with parasite clearance. The safety component was difficult to validate as all plants entering clinical trials were generally considered safe, so there was no clinical data on toxic plants.

**Conclusion:**

The RITAM score (especially the efficacy and safety components) can be used as part of the selection process for prioritising plants for further research as anti-malarial drug candidates. The validation in this study was limited by the very small number of available clinical studies, and the heterogeneity of patients included.

## Background

Over 1,200 plant species are reportedly used for the treatment of malaria and fevers worldwide, and are potentially important sources of new anti-malarial treatments [1]. As there are very limited funds for research on anti-malarial plants, it is important to prioritize plants for further research, notably for in depth laboratory studies and possibly clinical studies. The Research Initiative on Traditional Anti-malarial Methods (RITAM) was founded in 1999, and its objectives include to review current knowledge on traditional anti-malarial methods, to determine research priorities, to design optimal research methodologies, and to avoid replication of research [2].

Plants widely used as anti-malarials by traditional healers are significantly more active *in vitro* and/or *in vivo* against *Plasmodium sp* than plants which are not widely used, or not used at all, for the treatment of malaria [3-7]. A “retrospective treatment-outcome” study has been proposed to prioritize plants as anti-malarials, by studying clinical outcomes of patients who have used specified remedies for treating an episode of malaria [8]. This approach has proved to work well in Mali [9].

There already exists a wealth of published ethnobotanical and pharmacological studies on anti-malarial plants. However, this information has never been reviewed systematically and there is no standard method for doing so. Standard scores and methods have been developed for meta-analysis of studies of medical interventions and diagnostic tests [10]. There have been attempts at scoring plants according to basic ethnobotanical criteria (for example frequency of citation, or how widely a remedy is used [1,6] but these do not take into account all important factors such as the quality of studies or pharmacological information on efficacy and safety. Others have prioritized plants according to the selectivity index *in vitro*, corresponding to the ratio between cytotoxicity and activity against *Plasmodium falciparum*[*11*]. The first aim of this study was to design a standard score that could be used to prioritize traditional herbal remedies for further research based on objective criteria and systematic literature reviews, combining all available information from both ethnobotanical and pharmacological studies. The second aim was then to pilot this score and assess its ability to predict results of clinical trials for the few plant remedies that have been tested clinically for the treatment of malaria.

## Methods

### Design of the score

A “remedy” was defined as a specific preparation from a specific part of a plant species (for example, *Azadirachta indica* A. Juss. (Meliaceae) leaf decoction) or a defined mixture of parts from one or more plant species. After a comprehensive literature search, each remedy was given an overall score, composed of three components:

1: Frequency of citation in ethnobotanical studies (weighted according to quality of study, as detailed in Table 1)

**Table 1:** Quality score for ethnobotanical studies

Aspect (max score)	Criterion	Score
Type of study (2)	Primary Ethnobotanical study– original study consulted as source of information	+2
	OR: Primary ethnobotanical study, quoted in a review, but original data not available	+1
Botanical identification (3)	Plant collected and verified with informant	+1
	Voucher specimen in herbarium	+1
	Formal identification by botanist	+1
	Name given incorrectly	-1
Informant reliability (3)	Over 10 informants interviewed	+1
	>=2 informants mention use of the remedy for malaria	+1
	Informant(s) have experience of treating malaria	+1
Researcher reliability (2)	Same language as informants (i.e. information obtained directly without interpreter)	+1
	Detailed information recorded about remedies	+1
TOTAL SCORE (/10)		___/10 =

2: Efficacy *in vitro* (Table 2) and *in vivo* (Table 3)

**Table 2:** Laboratory efficacy score for *in vitro* antiplasmodial activity of crude extracts

IC_50_ (μg/ml) (best**)	Score	Level of activity
Not tested	0	Needs to be tested (priority to be determined according to other criteria)
< 2.0	+10*	Very good
2.0 - 5.0	+5*	Good
		This is the concentration range that is generally considered as active in screening programmes for anti-malarial activity, warranting bioassay-guided fractionation.
5.1 - 10	+3*	Good to moderate
		This range may reasonably be considered for bioassay-guided fractionation.
11 - 25	+2*	Weak
26 - 50	+1	Very weak
>100	-2	Inactive

** Aqueous extracts were priopritized but if these were not available, results from ethanolic extracts or methanolic extracts were used. Results from non-polar extracts such as dichloromethane and petroleum ether are too far removed from traditional preparations and were excluded.

*Add +1 if this level of activity is confirmed in more than one strain of P. *falciparum*, from a different continent or with different drug sensitivity, and +2 if confirmed in 2 or more strains.

**Table 3:** Laboratory efficacy score for *in vivo* antiplasmodial efficacy of crude extracts given to mice at the dose of 250 mg/kg/day (or lower)

% Inhibition	Score	Level of activity
Not tested	0	
100 - 90	+10	Very good activity
90 - 50	+5-9	Good to moderate
50 - 10	+1-5	Moderate to weak
0	-2	Inactive

3: Safety (Tables 4 &5)

**Table 4:** Safety scoring according to LD_50_

WHO classification	Score	Acute LD_50_ for rats (mg/kg body weight)			
Oral		Dermal	

Solid	Liquid	Solid	Liquid

Ia – Extremely hazardous	-10	≤5	≤20	≤10	≤40
Ib – Highly hazardous	-8	>5 – 50	>20 – 200	>10 – 100	>40 - 400
II – Moderately hazardous	-5	>50 – 500	>200 – 2000	>100 – 1000	>400 - 4000
III – Slightly hazardous	-2	>500 – 2000	>2000 – 3000	>1000 – 4000	>4000 - 6000
Unlikely to present acute hazard	+6	>2000	>3000	>4000	>6000
Not Tested	0				

(Source: ANNEX 2: WHO HAZARD CLASSIFICATION - ACUTE LD_50_ VALUES OF FORMULATED PRODUCTS. In: **THE GUIDEBOOK TO THE REGISTRATION OF PUBLIC HEALTH PESTICIDES AND REPELLENTS AGAINST VECTORS. http://app.nea.gov.sg/cms/htdocs/article.asp?pid=1211)**

**Table 5:** Safety score where LD50 has not been assessed

Safety	Score	Level of activity
Not evaluated	0	Needs to be tested (priority to be determined according to other criteria)

Reports of human toxicity	+2	Widespread and long-term use in humans with no reported toxicity
	-1	Reports of toxicity in humans after ingesting other parts of the same plant
	-2	Reports of mild toxicity in humans after ingesting the relevant plant part, or a mixture containing this part
	ELIMINATE	Reports of severe toxicity in humans after ingesting the remedy at a medicinal dose

Cytotoxicity: Activity Ratio (CAR) *in vitro* (CAR = IC_50_ for cytotoxicity, divided by IC_50_ for anti-malarial activity)	+10	≥1000
	+5	1000 > CAR≥100
	+2	100 > CAR≥ 50
	+1	50 > CAR≥ 10
	0	10 > CAR >= 2
	-5	CAR <2

Toxic chemical constituents	+2	Plant chemistry studied in depth, and no known toxic compounds have been found.
	-1	Toxic compounds found in a different plant part, or likely to be destroyed or evaporated in preparation of the remedy
	-3	Toxic compounds found in the relevant plant part, which are not likely to be destroyed in preparation

The total score was calculated for each remedy. The detailed scoring system was drafted and revised by a multidisciplinary working group including ethnobotanists, pharmacologists, phytochemists, clinicians and epidemiologists.

### Ethnobotanical score

This component was designed to take account of all citations of the remedy for the treatment of malaria or fever in ethnobotanical studies and historical sources, weighted by the quality of the studies. If the remedy of interest was a mixture, only citations of the whole mixture were included in calculating the score (not of individual components). However, if the remedy of interest was a single plant, citations including that plant in a mixture were included in calculating the score.

Each citation was required to meet the following inclusion criteria:

1.	Citations were preferred from primary ethnobotanical studies (whose quality could be assessed), quoting original data. Data from reviews or historical documents (manuscripts, materia medica) were included as long as the primary source could be identified and did not overlap with other citations.

2.	The ethnobotanical study was conducted in a malarious area, or in areas where malaria used to exist and there remained traditional knowledge of anti-malarial plants.

3.	The citation included information on the plant species, plant part, and method of preparation used.

The quality of each ethnobotanical citation was assessed according to the criteria in Table 1. A point was given for each of the criteria. If the publication made no mention of a particular criterion, no point was given for it. For each citation the points were added to a maximum of 10, and then divided by 10 to give a fraction (for example 10/10 = 1 ; 5/10 = 0.5). This fraction is the weighted score for the citation. Citations from reviews and historical sources (for example ancient herbals, pharmacopoeias) could not be scored in this same way, so were given a score of 0.1 for each citation. The weighted scores of all the citations of a particular remedy were then added to give the overall ethnobotanical score for that remedy. There was no maximum score. However if the score was 0 (*i.e.* the remedy is not traditionally used) the rest of the scoring system could not be used.

### Laboratory efficacy score

This component was intended to summarize the available information on efficacy of the remedy from pre-clinical pharmacological studies *in vitro* and *in vivo*. This scoring was done separately for different extracts, by plant part, extraction method and solvent used. Priority was given to extracts which mimicked most closely the traditional preparation. For example, if a methanolic extract was more active than an aqueous decoction, but the decoction was the traditional preparation, we gave precedence to the score for the decoction. Non-polar extracts which are very different from traditional preparations (e.g. dichloromethane, petroleum ether) were excluded. If the remedy of interest was a mixture, only results of laboratory studies of the whole mixture were included in calculating the score (not of individual components). For example, for the traditional remedy *Azadirachta indica* leaf decoction, the score would be based on the activity of an aqueous decoction of *A. indica* leaf (NOT a methanol or ethanol extract of the leaves, or the oil from the seeds). Studies were discounted if there was no adequate information on botanical identification of the plant.

When there were several results for one type of extract, from studies of adequate quality, the score was given according to the best result (i.e. lowest IC_50_), as in table 2. Extra points were available if the activity had been confirmed in more than one strain of *P. falciparum* with different drug sensitivities and from different geographical endemic areas. This score was adapted from a previous score [12] which had been designed for assessing fractions rather than crude extracts. Thus the score has a maximum value of +12 for the *in vitro* component. For the purposes of this review only *in vitro* tests on intraerythrocytic parasites were considered, as the focus was on plants used for treatment (rather than prevention) of malaria.

If the remedy had also been tested *in vivo* by the Peters’ 4-day suppressive test [13] in animal models (and administered orally), additional points were added to the efficacy score as in Table 3: one point was given for each 10% of inhibition. When these components were added, the maximum possible efficacy score was +22, and the minimum possible score was –4.

Often extracts are given by the intra-peritoneal route, and many are efficacious because bioavailability is often better than by the oral route. However such results were excluded because the intra-peritoneal route is never used traditionally and results cannot be applied to oral administration. Furthermore there is a greater risk of toxicity.

The arbitrary dosage of ≤ 250mg/kg/d was chosen as an inclusion criterion for the treatment of mice according to the Peters’4-day suppressive test. However, many studies reported either only ED_50_ (which is the effective dose reducing the parasitaemia by 50% in comparison with untreated controls) or tests with other doses from 25 mg/kg/d to 1000 mg/kg/d. Doses of >250mg/kg/d, which are probably too high to use in practice, did not score any points. The pharmacological response may also be affected by the murine *Plasmodium* species and strain used in a particular test (chloroquine resistant or sensitive *Plasmodium berghei*, *Plasmodium vinckei*, *Plasmodium yoelii*, *Plasmodium chabaudi*) [14-16].

### Safety score *(maximum score = +6, minimum = -10)*

This component summarized available knowledge on safety of the remedy. If LD_50_ (which is the dose lethal for 50% of the animals) data is available in the literature for some remedies or plant parts, the score is given according to this (see Table 4). The WHO classification suggests this should be done in rats, but for the present score data was used from rats or mice, whichever was available. If LD_50_ data was not available, the score in Table 5 was used to summarize information from any reports of human toxicity, cytotoxicity tests, and phytochemical analysis.

### Overall score

The overall “RITAM score” was the sum of each of the above components, and was used to rank remedies as a way of prioritising them for further research. An example of how the score was calculated is presented in Table 6:

**Table 6:** Calculation of the RITAM score for *Vernonia amygdalina* leaf decoction

Component of Score	Parameter	Value	Reference	Score
Ethnobotanical	Citation scores (*see table*1*for details of calculation*)		[40]	0.5
			[41]	0.6
			[42]	0.9
			[43]	0.6
			[44]	0.6
			[45]	0.5
			[46]	0.5
			[47]	0.8

Efficacy	IC_50_*in vitro*	76.7 µg/ml	[48]	0
	% inhibition *in vivo*	62.7%	[49]	6

Safety	LD_50_	3320mg/kg	[50]	6

TOTAL				17

Overall RITAM score for a remedy =

	Ethnobotanical score (*no maximum score*)

*+* Laboratory Efficacy score *(maximum score = +22, minimum score = -4)*

*+* Safety score *(maximum score = +6, minimum = -10)*

The component scores were listed as well as the total, to enable searching and selection according to different criteria. For example, natural product chemists may be less interested in the safety score, as an isolated compound may be less toxic or could be modified chemically to reduce toxicity.

### Validating the score

A systematic literature review was conducted of clinical trials of anti-malarial plants [17]. All published clinical trials of herbal anti-malarials were identified through systematic searches of the MEDLINE, EMBASE and CABI Global Health databases, and by consulting experts for unpublished data. We then applied the following criteria to select trials for inclusion in this analysis:

1. A traditional herbal remedy (rather than a modern combination of traditional plants, for which there would be no reports in the ethnobotanical literature)

2. Controlled trials or cohort studies including at least 20 patients

3. Parasite clearance at day 7 ascertained by a reliable method (with two microscopists and/or examining 100 high power fields of a thick film before declaring a film as negative)

4. Symptom clearance at day 7 reported, and/or Adequate Clinical Response (ACR) at day 14.

However, parasite clearance and symptom clearance were also included as they are the most often cited in studies, although they may not be the most appropriate measures of effectiveness. ACR is the outcome recommended by the RITAM guidelines [18], based on WHO guidelines [19]. Incidence and severity of side-effects were also assessed as important secondary outcomes. ACR is defined as absence of parasitaemia on day 14 irrespective of axillary temperature, without previously meeting any of the criteria of early or late treatment failure; or axillary temperature <37.5°C irrespective of the presence of parasitaemia, without previously meeting any of the criteria of early or late treatment failure. Early treatment failure is defined as development of danger signs on day 1, 2 or 3 in the presence of parasitaemia; or axillary temperature ≥37.5Â°C on day 2 with parasitaemia > day 0 count; or axillary temp ≥37.5Â°C on day 3 with parasitaemia. WHO guidelines also count afebrile patients with parasitaemia on day 3 ≥25% of count on day 0 as early treatment failures, but these are not included in the modified RITAM definition. Late treatment failure is defined as development of any danger signs or signs of severe malaria, or axillary temperature ≥37.5Â°C, in the presence of parasitaemia on any day from day 4 to day 14, without previously meeting any of the criteria of early treatment failure.

For the same remedies, the RITAM score was calculated following a systematic literature search of the same databases for ethnobotanical and pharmacological studies of anti-malarial plants. Experts were also contacted for other sources of ethnobotanical and pharmacological studies. Over 100 ethnobotanical studies and 52 pharmacological studies, as well as existing literature reviews were consulted [20].

Spearman rank correlation coefficients (r_s_) [21] were calculated for correlation between RITAM scores and clinical outcomes (see Table 7). The Kendall partial rank correlation coefficient [21] was used to adjust for the age of the patients included in the studies. A sensitivity analysis was conducted by only including patients aged 12 years and over with baseline parasite counts of 500 per µl and over, which in one case involved re-analysis of the raw data (see Table 8).

**Table 7:** RITAM Scores compared to results of good quality clinical trials in uncomplicated falciparum malaria

Remedy	RITAM score				Clinical results				Study characteristics			Ref

	Overall	Ethnobotanical	Efficacy	Safety	Parasite clearance d7 (%)	Fever clearance d7 (%)	Side effects (%)	ACR d14 (%)	N of patients	Mean age (yrs)	Geometric mean parasitaemia d0	
*Artemisia annua* L. (Asteraceae) aerial parts infusion	19.9	0.9	13	6	74%	86%			72			[27]
*Vernonia amygdalina* Delile (Asteraceae) leaf decoction	17.0	5.0	6	6	32%	67%	65%	67%	33	29	5393	[28]
*Argemone mexicana* L. (Papaveraceae) leaf decoction	14.9	1.9	7	6	21%	74%	17%	73%	231	10	1746	[35,51]*
*Cochlospermum planchonii* Hook. f. ex Planch. (Bixaceae) root decoction	6.0	2.0	1	3	52%	62%	46%		46	23	11191	[29]
*Combretum micranthum* G. Don (Combretaceae) mixtures	0.5	0.5	0	0	14%	79%	13%	28%	78	4	3874	[32]

* Data from these two studies were pooled for the analysis

**Table 8:** Sensitivity analysis including only patients aged >12 years with baseline parasitaemia of >500 per mcl

Remedy	RITAM score				Clinical results				Study characteristics		Refes

	Overall	Ethnobotanical	Efficacy	Safety	Parasite clearance d7 (%)	Symptom clearance d7 (%)	Side effects (%)	ACR d14 (%)	N of patients	Baseline parasitaemia (gm)	
*Artemisia annua* aerial parts infusion	19.9	0.9	13	6	74%	86%			72		[27]
*Vernonia amygdalina* leaf decoction	17.0	5.0	6	6	32%	67%	65%	67%	33	5393	[28]
*Argemone mexicana* leaf decoction	14.9	1.9	7	6	50%	88%	13%	94%	16	3162	[30]*
*Cochlospermum planchonii* root decoction	6.0	1.0	2	3	52%	62%	46%		46	11191	[29]

*Subgroup analysis including only patients with the defined characteristics

## Results

Ten herbal remedies were identified that have undergone clinical trials published in the literature, meeting our inclusion criteria. Trials of only three remedies included “adequate clinical response” as an outcome, so it was not possible to calculate the correlation between RITAM scores and this outcome. The quality of the clinical trials was variable; in particular five trials did not specify the methodology for ascertaining parasite clearance. These are likely to overestimate parasite clearance, so they were eliminated from further statistical analysis. The trials excluded for this reason were those of *Caesalpinia crista* (Fabaceae) seed powder [22], *Cinchona* (Rubiaceae) bark extract [23], *Dichroa febrifuga* (Hydrangeaceae) root decoction [24], *Cochlospermum tinctorium* (Bixaceae) root decoction [25], and *Cryptolepis sanguinolenta* (Apocynaceae) root infusion [26].

The five trials using adequate methods for measuring parasite clearance are shown in Table 7. Spearman rank correlation identified that parasite clearance was correlated with the efficacy score (r_s_ = 0.6) and with average age of the patients (r_s_ = 0.7). The analysis was then stratified according to age. There were too few studies of children under five years to permit any meaningful analysis in this age group. Data was available from four studies (or subsets thereof) of patients aged 12 years and above [27-30], although only two of these used ACR as an outcome measure. In this subset parasite clearance correlated better with the efficacy score (r_s_ = 0.7). The ethnobotanical score did not correlate with parasite clearance (r_s_ = 0), but there was a slight correlation with symptom clearance (r_s_ = 0.5). Too few clinical studies reported on the incidence of side-effects to be able to calculate a correlation with the safety score. Almost all of the plants selected in this validation had a high safety score, as would be expected. None of the trials reported any serious adverse effects.

## Discussion

This is a first attempt to devise and pilot a scoring system to prioritize anti-malarial herbal remedies for further research, based on existing ethnobotanical data, and laboratory data on efficacy and safety. The overall score for most promising remedies was over 14, showing good results in all domains. However combining the scores can also have disadvantages. *Cinchona* (which is highly effective, and the source of quinine, which can be toxic [31]) scored 6.5 overall (ethnobotanical = 3.5; efficacy = 8; safety = -5) which was the same score as the safe but ineffective topical Shea butter (ethnobotanical = 0.5; efficacy = 0; safety = 6) [32].

The evaluation of the proposed RITAM score is limited by the paucity of good quality published clinical trials of herbal anti-malarials. Despite an exhaustive literature search, clinical trials of only ten remedies were identified, only five of which had used good quality methods for evaluating parasite clearance, and only three of which had recorded ACR as an outcome. Even in some of these the preparation and dose may not have been optimal.

Definition of clinical outcome is of central importance to this evaluation. Prevention of severe malaria is in fact the desired effect, and can be achieved without total parasite clearance [30,33], but large numbers of patients are needed in order to detect differences in this outcome, so it is not commonly used. ACR was devised as a surrogate measure but its definition is complex, and may be interpreted slightly differently in different studies [34]. Parasite clearance is a simpler outcome which should have the same definition in different studies, but its relevance is debatable in high transmission areas where reinfection occurs rapidly [33]. Several clinical studies reported significant declines in parasite counts although total clearance was not achieved [25,35]. The accurate assessment of parasite clearance requires high quality methods, which is why clinical studies not reporting such methods were excluded. Almost all of the trials reported symptom clearance in 60% or more of the patients, which suggests that traditional medicines are at least effective at relieving symptoms. The definition of “symptom clearance” also varied between studies so the figures reported are not necessarily comparable. Similarly, methods for ascertaining side-effects varied between trials, so the incidence figures are not comparable between trials. A checklist of possible side-effects [28] will inevitably generate a higher incidence of reports than asking an open question about side-effects, which was used in some other studies [35]. Some of the symptoms reported may well have been due to the disease rather than to the treatment [29]. In some of the clinical studies it is not clear whether patients were even asked about possible side-effects.

Clinical recovery and parasite clearance depend not only on the efficacy of the remedy but also on the level of immunity of the patient. All of the clinical studies took place in areas of intense seasonal transmission in sub-Saharan Africa, although the transmission season may have been shorter in the area where Bugmann’s study took place, so levels of immunity may have been lower there [32]. Age is one of the major confounders and explains at least some of the differences between the studies. Several studies included only patients above the age of 12 [28,29] or 18 [27] and these tended to have better parasite clearance and adequate clinical response rates than the studies including younger children [30,32,35].

The correlation of the laboratory efficacy score with parasite clearance suggests that pre-clinical studies are useful predictors of clinical efficacy. There may be a publication bias because poor results are less likely to be published. However neither *in vitro* nor *in vivo* tests predicted all clinically useful remedies. *Vernonia amygdalina* (Asteraceae) had low *in vitro* activity but good *in vivo* activity (table 6). *Argemone mexicana* (Papaveraceae) did not show any activity in animals, although there was clear evidence of activity *in vitro* and in humans [30]. A better correlation might be obtained by testing the anti-malarial activity *in vitro* of the serum of healthy volunteers having ingested the remedy [36], but this method has not been widely used. This would avert the problem of contaminating compounds such as saponins that complicate traditional testing of extracts *in vitro*.

The ethnobotanical score did not correlate with parasite clearance, but did correlate weakly with symptom clearance. This supports the view that traditional healers select plants which act on the symptoms, although not necessarily on the underlying cause of the disease. One limitation of the score is that for some plants the bulk of the ethnobotanical information is in documents which are unpublished or which are not included in international databases. A case in point is *Artemisia annua* (Asteraceae) which had the highest efficacy score and the best parasite clearance, but for which there is almost no ethnobotanical information in the international literature, so it had a very low ethnobotanical score. It was selected by the Chinese because of information in traditional Chinese texts, which are not catalogued in standard international databases. In fact another species (*A. apiacea* Hance) was used preferentially in ancient Chinese medicine, but it has never undergone clinical trials [37]. Inclusion of national and local databases (especially Chinese) may improve the validity of the ethnobotanical score, but in practice this is difficult to do. Another concern is the influence of geographic range on the ethnobotanical score. Plants with a small geographic distribution do not have the opportunity to be cited in many studies. *Cryptolepis sanguinolenta* is one such plant, which is reported only in Ghana and in the Congo, but with a strong local reputation. Strong ethnomedical evidence, such as from a retrospective treatment-outcome study [8,38], is probably a better predictor of efficacy than the number of citations. A revised score might take into account how extensively the plant is used across its distribution in malarious regions.

The safety component of the score was difficult to evaluate as few clinical trials contained quantitative information on incidence of side-effects, and furthermore clinical trials would only be done on plants which are well known to be non-toxic. The remedy with the lowest safety score (-5) was *Cinchona* bark, because of the reports of mild side-effects from use in humans [31], and because it contains potentially toxic alkaloids (including quinine). However, the clinical trials report that the incidence of side-effects from the bark was no greater than with the use of pure quinine [31]. Therefore the presence of toxic compounds should perhaps be given less weight because toxicity always depends on dose and many effective medicines are toxic when excessive doses are given. The safety score is important to ensure toxic remedies are filtered out and not taken forward into clinical research, but there is a risk that it may screen out some of the most effective remedies that are toxic only at doses higher than the therapeutic dose, or have only mild and usually tolerable side-effects (such as *Cinchona*). For this reason it may be preferable to use a therapeutic index obtained *in vivo* by the ratio LD_50_/ED_50_. It is possible that a highly active plant would also be highly toxic, and so may receive a positive overall score. However it is very unlikely that an extremely hazardous plant would survive the test of time as a traditional medicine (or indeed that those taking it would survive or encourage others to use it), and this score is only intended for plants which are used as traditional medicines. Although it was not possible to validate the safety component of the score, safety is a very important consideration for prioritization of plants. In the absence of anything better we suggest that the safety score should be used as part of the selection process for prioritizing plants.

Another drawback of the score is that it is difficult to evaluate complex remedies which contain several plants. Most ethnobotanical studies report on uses of single plants rather than combinations, so that the ethnobotanical score would be low for such remedies. It is also rare for such combination remedies to be tested as such *in vitro* and *in vivo*. The only exception we found was “Malarial”, a combination of three plants used in Mali, which had undergone preclinical parasitological and safety tests prior to clinical trials and registration as an “improved traditional medicine” [39].

Although this scoring system was developed specifically to prioritize anti-malarial plants, it could be modified as a way of prioritizing plants for clinical trials on other diseases, although it would need to be validated again using relevant trials. The ethnobotanical component might be expected to be useful for diseases which are easily recognized traditionally, for example intestinal worms, dysentery, and skin ulcers. It would not be useful for diseases which have been newly discovered or which cannot be diagnosed without modern medical equipment (such as HIV/AIDS or Chagas disease). The efficacy component could however be adapted for any disease for which laboratory models exist, as a way of prioritising among many plants tested. The safety component could be applicable for any remedy (although we must stress that this part of the score could not be validated in our study).

## Conclusions

The overall RITAM score can be used as part of the selection process for prioritizing anti-malarial plants for future research, alongside other factors such as ease of cultivation and preparation. In particular the laboratory efficacy component of the score correlated with parasite clearance in good quality clinical trials, and so can be used as one way to prioritize and rationalize the selection of herbal remedies for future clinical studies. The ethnobotanical score was not useful because the score was low for plants whose use is mainly reported in traditional texts, which cannot easily be accessed from modern databases, and for plants whose distribution is localized. The safety score is important but we were unable to evaluate this fully because all of the plants taken into clinical trials and published were relatively non-toxic. The validation in this study was limited by the very small number of available clinical studies, and the heterogeneity of included patients. More clinical studies of herbal anti-malarials are needed, and as these become available it should be possible to improve the scoring system and its validation.

## Competing interests

The authors declare that they have no competing interests.

## Authors' contributions

MLW drafted the ethnobotanical and safety components of the score, and PR and FBV designed the laboratory efficacy components. All authors helped to refine these scoring systems. MLW and DF conducted the literature searches and calculated scores for included remedies. MLW assessed clinical trials, conducted the statistical analyses, and wrote the first draft of the paper. All authors read and contributed to the article, and approved the final manuscript.
